# Opening and closure of intraventricular neuroendoscopic procedures in infants under 1 year of age: institutional technique, case series and review of the literature

**DOI:** 10.1007/s00381-020-04895-x

**Published:** 2020-09-27

**Authors:** M. D. Cearns, M. Kommer, A. Amato-Watkins, E. Campbell, T. Beez, R. O’Kane

**Affiliations:** 1 Department of Paediatric Neurosurgery, Royal Hospital for Children, Glasgow, UK; 2 Department of Neurosurgery, Medical Faculty, Heinrich-Heine-University, Düsseldorf, Germany

**Keywords:** Hydrocephalus, Endoscopic third ventriculostomy, CSF leak

## Abstract

**Purpose:**

Intraventricular neuroendoscopic techniques, particularly third ventriculostomy, are employed increasingly in the management of infantile hydrocephalus. However, surgical access to the ventricular cavities is associated with a risk of post-operative cerebrospinal fluid (CSF) leak. Here, we describe a structured, multi-layered approach to wound opening and closure which aims to maximise the natural tissue barriers against CSF leakage. We present a series of patients undergoing this technique and subsequently review the literature regarding opening and closure techniques in paediatric intraventricular neuroendoscopic procedures.

**Methods:**

We performed a retrospective case series analysis of patients under 1 year of age who underwent intraventricular neuroendoscopic procedures in a single institution over a 5-year period. Patients were identified from an institutional operative database, and operation notes and clinical records were subsequently reviewed.

**Results:**

28 patients fulfilled the inclusion criteria for this study. The mean age at operation was 9 weeks. 27 patients underwent endoscopic third ventriculostomy whilst 1 underwent endoscopic septostomy, and all patients underwent our structured, multi-layered opening and closure technique. Follow-up ranged from 4 months to 5 years. There were no cases of post-operative CSF leak, infection or wound breakdown. 12 patients remained shunt-free at the last follow-up, with the remaining 16 requiring shunt insertion for progressive hydrocephalus at a mean of 24 days post-operatively.

**Conclusion:**

Various methods aiming to prevent post-operative CSF leak have been reported in the literature. We propose that our institutional technique may be of benefit in minimising this risk in infants undergoing endoscopic third ventriculostomy and similar intraventricular neuroendoscopic procedures.

## Introduction

Intraventricular neuroendoscopic techniques are utilised increasingly in the management of hydrocephalus, including in infants. This is principally in the form of endoscopic third ventriculostomy (ETV), a minimally invasive technique which allows excellent visualisation of anatomical structures and can potentially delay or avoid the need for a cerebrospinal fluid (CSF) shunt [1–4]. CSF leakage is a well-recognised post-operative complication of ventricular surgery, with morbidity resulting from associated superficial and intracranial infection, including wound breakdown, meningitis and ventriculitis [5]. Indeed, this risk may be higher in hydrocephalic infants, given the thin cerebral mantle separating the ventricles from the surgical entry point. Such complications can be life-threatening or can cause recurrence of the hydrocephalus with profound neurological and cognitive developmental consequences for infants. A recent meta-analysis reported a 5.8% CSF leak rate following ETV in children [6].

Previous practice in our institution was a conventional approach for ETV in infants involving a linear skin incision with ventricular access either through an underlying burr-hole or via the open anterior fontanelle. Following endoscopy, the dura and scalp layers were closed. However, following three cases of CSF leak in such patients in our institution, we evaluated our practice and moved to a structured, multi-layered approach to opening and closure of the wound. This is one of the various published techniques to minimise the risk of post-operative CSF leak, and it aims to maximise the natural tissue barriers during closure. Here, we present a detailed description of the technique we employ in the context of a case series of 28 patients who underwent this technique. Following this, we review the methods previously published in the literature which aim to minimise the risk of CSF leak following intraventricular neuroendoscopic surgery in infants.

## Methods

We performed a retrospective case series analysis of patients under a year of age who underwent an intraventricular neuroendoscopic procedure in our institution between February 2015 and January 2020. Patients were identified from a departmental operative database, and operation notes and clinical records were reviewed.

### Opening technique (see Fig. 1)

A U-shaped frontal incision is fashioned behind the hairline, centred on the midpupillary line. This is achieved by scoring the skin with a blade and then completing the incision with monopolar diathermy (Colorado microdissection needle, Stryker®). The medial curve of the wound may cross the anterior fontanelle, so care is taken not to breach the dura. The pericranium is opened in the same pattern and peeled off the skull and fontanelle using saline-soaked gauze. This is used to maintain pericranial integrity and prevent drying out, which may impede closure. The bone is elevated away from the fontanelle and underlying dura, and either levered or broken as a greenstick-like fracture at the anterior limit of the operative site. The dura is opened in a reverse U-shaped pattern and also kept from drying out. The cerebral cortex, now exposed, can be entered via a small corticotomy with a Dandy cannula prior to endoscopic access to the ventricle.

**Fig. 1:**
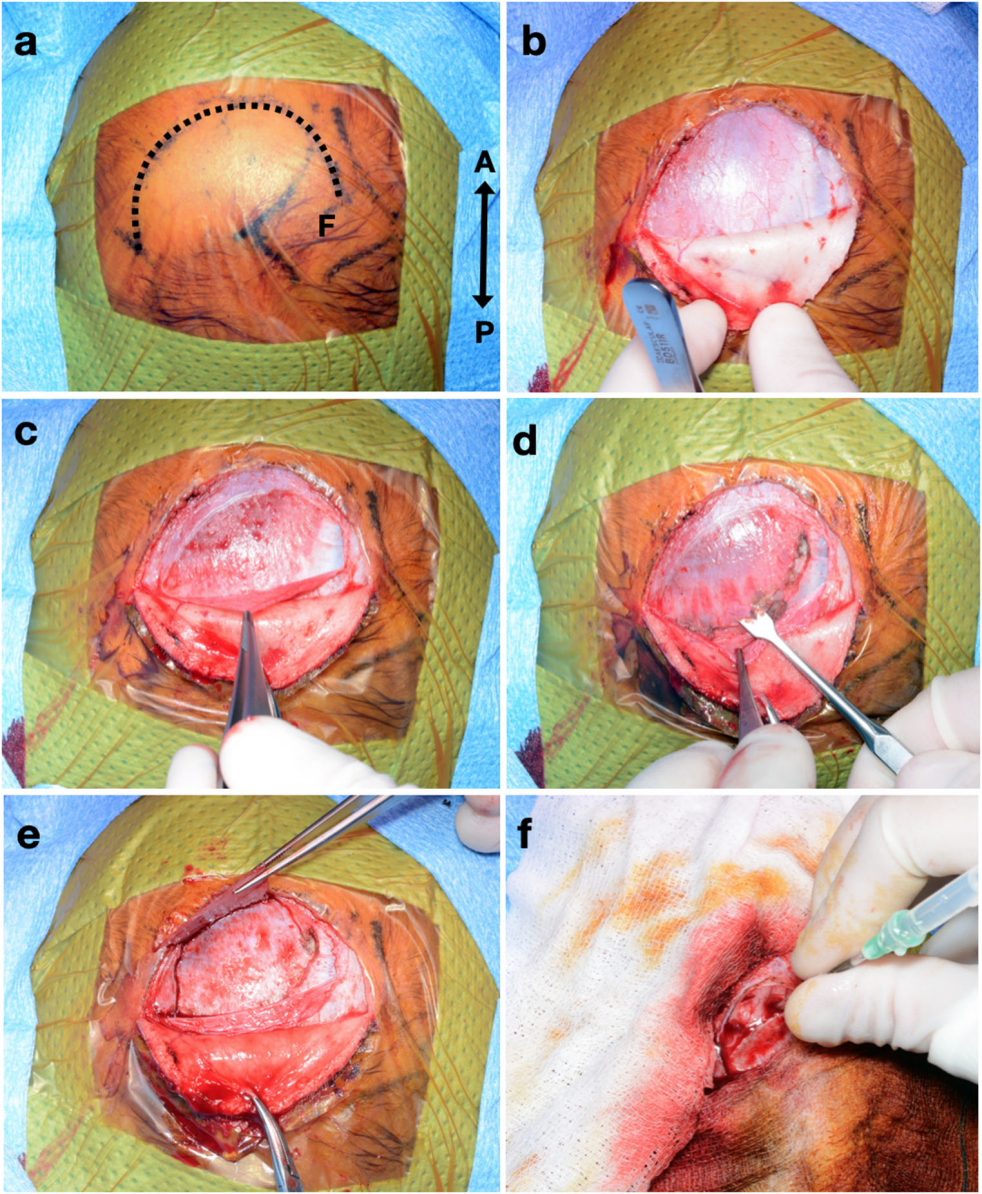
Wound opening technique (all photographs with same orientation; A, anterior; P, posterior). **a** Skin incision (dotted line), centred on midpupillary line and extending to midline over anterior fontanelle (F; boundaries indicated with skin marking pen). **b** Elevation of skin flap. **c** Elevation of pericranium (held with forceps). **d** Separation of bone from dura of fontanelle. **e** Greenstick-like fracture of bone (held with forceps) to reveal underlying dura. **f** Corticotomy with Dandy cannula following reverse U-shaped durotomy (dural edges visible)

### Closure technique (see Fig. 2)

The goal of this technique is as watertight a closure as possible, and each layer opened is given individual consideration for this. The corticotomy edges are apposed and glued with a TISSEEL (Baxter®) fibrin sealant. The dura is closed as a continuous layer with 4/0 PDS. The bone is placed back in its original position, tacked with several interrupted 2/0 Prolene sutures and the edges sealed with TISSEEL. The pericranium is closed, ideally continuously with 4/0 PDS, but failing this then in an interrupted fashion. Again, TISSEEL is applied along the edges. The galea is closed with continuous 3/0 Vicryl and finally the skin with continuous 4/0 Monocryl. Post-operatively, the patient is nursed at 30° head up and standard neurological observations are employed.

**Fig. 2:**
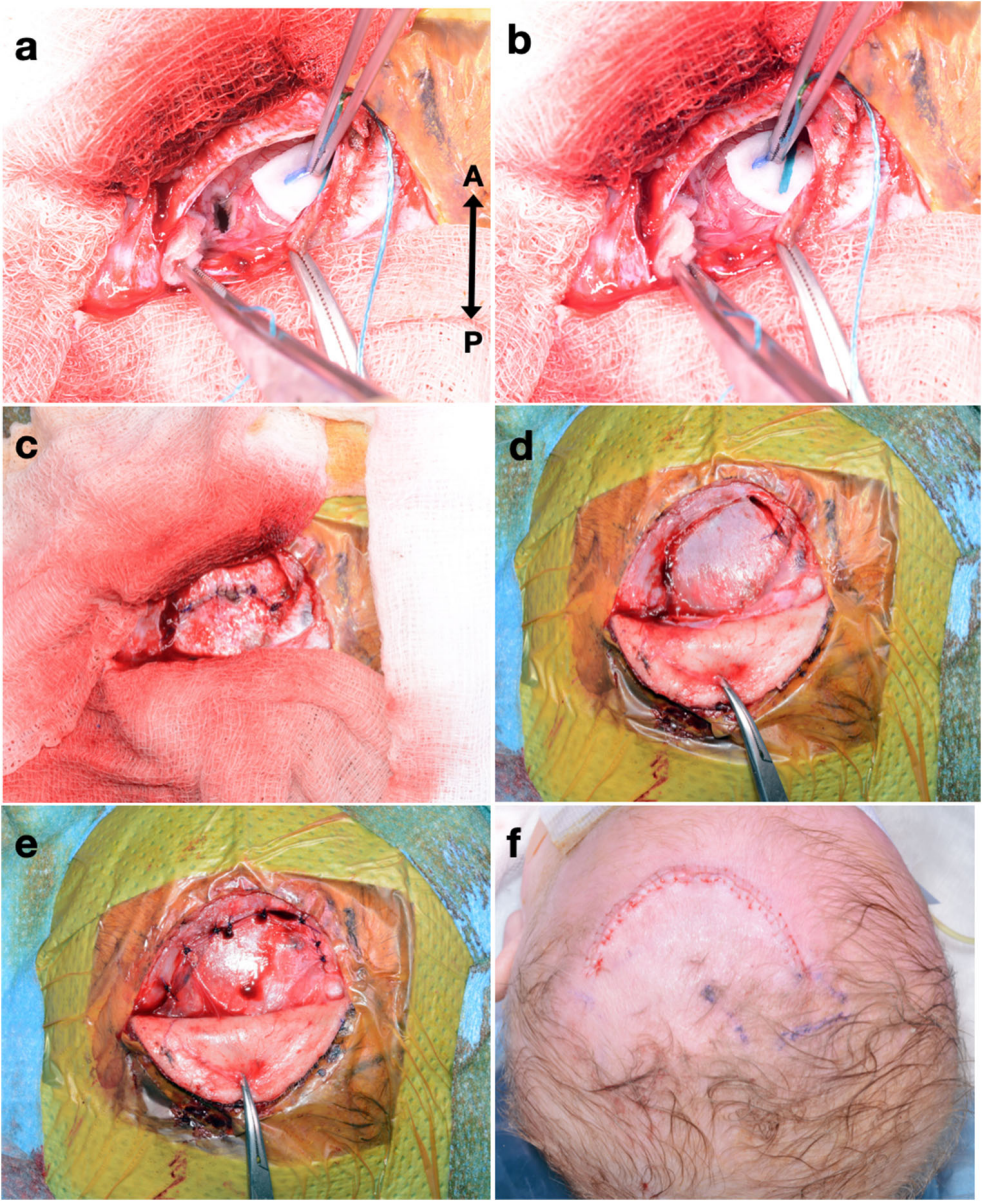
Wound closure technique (all photographs with same orientation; A, anterior; P, posterior). **a** Corticotomy visible following removal of an endoscope. **b** Corticotomy edges apposed with patties and TISSEEL (Baxter) fibrin sealant. **c** Dura closed, continuous 4/0 PDS. **d** Bone replaced with several interrupted 2/0 Prolene sutures and TISSEEL. **e** Pericranium closed, continuous or interrupted 4/0 PDS. **f** Following continuous galeal closure (3/0 Vicryl), skin closed with continuous 4/0 Monocryl

## Results

28 patients (16 male, 12 female) were identified for this study, of whom 27 underwent ETV and 1 underwent endoscopic septostomy. Additional procedures performed included choroid plexus cauterisation (14), cyst fenestration (7), aqueductoplasty (3), septal fenestration (2) and evacuation of concurrent intraventricular haemorrhage (1). All patients underwent our multi-layered opening and closure technique. The mean age at operation was 9 weeks (range: 5 days–6 months). The length of follow-up ranged from 4 months to 5 years. There were no cases of post-operative CSF leak, infection or wound breakdown in this series. 12/28 patients remain shunt-free at last follow-up. Of the remaining 16, shunt insertion occurred on average 24 days following their neuroendoscopic procedure (range: 2 days–10 weeks). In all cases, the indication for shunt insertion was progressive hydrocephalus, with no evidence of CSF leakage.

## Discussion

Given the potentially devastating effects of intracranial infection associated with post-operative CSF leakage, it is important to employ technical approaches that may reduce the risk of this complication. These can be considered according to management of each anatomical layer from the skin of the scalp through to the cerebral cortex, beginning with the planning of an appropriate technique for incision and opening. We would argue that the conventional placement of a linear incision immediately overlying a burr-hole generates a potential linear route for the post-operative leakage of CSF. This can be avoided by means of a curvilinear skin incision with reflection of a flap, which allows the bony defect to be spatially removed from the point of weakness in the skin [7, 8]. In order to further bolster this technique, the galea can be exposed following initial skin incision and incised more anteriorly, along with the pericranium, to raise a separate galeal-pericranial flap. This further maximises use of natural tissue barriers as the flap can be closed separately from the skin [7]. Alternatively, the pericranium can be closed separately from the galea and the skin as its own distinct anatomical layer, a method we employ in our institutional technique [9].

The approach to bony opening of the calvarium is likely to play an important role. Some groups advocate plugging of the burr-hole with absorbable gelatin sponge, or reinforcement of the burr-hole with a bone graft or metal covering [9]. Alternatively, methods can be employed which avoid a bony defect entirely. One such method is an approach through the open anterior fontanelle, which in young infants has the benefit of avoiding disruption to the growing calvarium [10]. However, with this technique, there is no bone layer separating the site of potential CSF leakage from the skin, and therefore, it does not maximise natural tissue barriers. An alternative means of retaining a protective layer of bone whilst avoiding a burr-hole defect is to incorporate a minicraniotomy [8, 11]. Although this is felt to be an effective way to minimise the risk of CSF leak, it has been criticised as being unnecessary for the purposes of endoscopic ventricular access, given that it involves more significant bony removal and can be challenging in young infants with very thin dura [7, 10]. However, one available method in young infants is the greenstick-like fracture, which we employ in our institutional technique. This has been previously reported as a way of utilising the open anterior fontanelle to separate the frontal bone from the dura and elevate it anteriorly [8]. It generates a bone flap which remains attached at its anterior border and avoids placement of a burr-hole. The shape and orientation of this bone flap create a further barrier to CSF leakage as well as abolishing any bony defect once it is replaced. We have found that tacking sutures can be placed through the edges of the bone in such young infants to return it to its original position.

Finally, consideration must be given to the deepest anatomical layers involved: the dura mater and the site of the corticotomy following removal of the endoscope. The dura has not traditionally been closed in endoscopic procedures and indeed dural closure can be challenging if the procedure is carried out through a burr-hole only. Cinalli proposes this as one of the benefits of minicraniotomy and advocates continuous watertight dural closure [11]. Yadav and colleagues report moving to linear dural incision and formal closure in infants with gross hydrocephalus and a thin cortical mantle, in whom the risk of CSF leak was deemed to be greater [12]. Dural closure may be bolstered by the use of dural sealants [9]. An alternative approach utilised by Sufianov and colleagues was to minimise disruption to the dura by use of a miniature sialendoscope, which was used to pierce the dura, cortex and ventricle directly. Neither formal dural opening nor closure was performed and they report no post-operative CSF leakage in 64 paediatric cases, which they attribute to avoidance of dissection and coagulation of the dura [10]. In terms of the corticotomy, the site from which any post-operative CSF leakage will arise, the use of compressed gel foam has been reported to reduce the risk of leakage [7, 13]. In this series, we carefully use TISSEEL to seal the corticotomy. However, care must be taken to prevent these products from passing into the ventricular cavity and causing obstruction, and it has been proposed that over-plugging of the cortical tract with gel foam may in fact induce CSF leakage via a ‘wick’ effect [7].

We feel that our institutional technique provides a structured, multi-layered approach to wound opening and closure that maximises the natural barriers against CSF leakage and has led to improvements in CSF leak rate in our institution. It incorporates some of the technical approaches previously described in the literature, although we believe that its utility also lies in its being anatomically well structured and technically reproducible. We acknowledge that the number of patients is relatively low; however, in the context of our experiences with a conventional approach, we feel that an absence of post-operative wound complications in this series is of value. The formal closure of multiple layers and the use of watertight continuous closures where possible are likely to have played an important role. We advocate the use of TISSEEL fibrin sealant at multiple stages in the closure process, as this appears to add downward pressure between layers and contributes to a water-resistant closure. Furthermore, using a greenstick-like fracture of the calvarium allows the free edge of the bone to be spatially removed from both the skin incision above and the durotomy below, which may help avoid a potential linear route for any CSF leakage. We hope that this technique may be useful to others aiming to reduce the rate of CSF leak in patients undergoing intraventricular neuroendoscopic surgery, and we continue to follow the pattern of our results.

## Data Availability

All patient data are anonymised; all references are supplied.
